# Two-year follow-up of 90 children with autism spectrum disorder receiving intensive developmental play therapy (3i method)

**DOI:** 10.1186/s12887-022-03431-x

**Published:** 2022-06-28

**Authors:** Eloïse Brefort, Yann Saint-Georges-Chaumet, David Cohen, Catherine Saint-Georges

**Affiliations:** 1 Maison Médicale, May-sur-Orne, France; 2 Bioredac, 97 Grande rue, 78240 Chambourcy, France; 3 Department of Child and Adolescent Psychiatry, Hôpital de la Pitié-Salpêtrière, Sorbonne University, 75013 Paris, France; 4 Institut des Systèmes Intelligents et de Robotiques, CNRS UMR 7222, Sorbonne University, Paris, France

**Keywords:** ASD, 3i method, CARS, ADI-R, Developmental intervention

## Abstract

**Background:**

The Intensive, Interactive, and Individual (3i) intervention approach aims to decrease the severity of autism spectrum disorder (ASD) using intensive developmental play therapy (3i). We performed a retrospective study of 90 children who were enrolled for 2 years in the 3i approach to assess changes and predictors of changes in ASD severity at follow-up (FU).

**Methods:**

The ASD severity of all patients (*N* = 119) who began 3i intervention between 2013 and 2018 was systematically measured using the childhood autism rating scale (CARS) and autism diagnosis interview-revised (ADI-R). Among them, 90 patients (mean age 5.6 ± 3.7 years) had a second assessment at the 2 year FU. CARS and ADI-R scores after 2 years of 3i intervention were compared to baseline scores using paired student’s t-tests. We used multiple linear regression models to assess the weight of baseline variables (e.g., age, oral language, sex, treatment intensity) on changes at the 2 year FU.

**Results:**

Mean CARS and ADI-R subscores (interaction, communication, repetitive behaviour) decreased significantly by 20, 41, 27.5 and 25%, respectively (effect sizes: d > 0.8). Moreover, 55 and 46.7% of participants switched to a lower category of ASD severity based on the CARS scale and ADI-R interview, respectively. Multiple linear models showed that (i) a higher treatment intensity (more than 30 h per week) was significantly associated with a greater decrease (improvement) in the ADI-R interaction score; (ii) patients categorized as verbal subjects at baseline were associated with a better outcome, as ascertained by the CARS, ADI-R interaction and ADI-R communication scores; and (iii) older patients were significantly associated with a greater decrease in the ADI-R interaction score. However, we found no impact of sex, severity of ASD or comorbidities at baseline.

**Conclusion:**

This study performed on 90 children suggests that 3i therapy may allow for a significant reduction in ASD severity with improvements in interaction, communication, and repetitive behaviours. A study using a control group is required to assess the efficacy of 3i play therapy compared to other interventions.

**Supplementary Information:**

The online version contains supplementary material available at 10.1186/s12887-022-03431-x.

## Introduction

Autism spectrum disorders (ASDs) are neurodevelopmental disorders that impairsocial relationships and communication. These dysfunctions occur from birth and produce abnormalities in oral and/or nonverbal communication, social interactions and restricted interests in the first years of life. Although, the number, type, and severity of symptoms, as well as the age of their onset, vary from person to person, these difficulties in behaviour, communication and social interaction typically begin before the age of 3 years. In 2013, the DSM-5 [1] described the frequent sensory or perceptual impairments of children with ASD that may lead to hypersensitivity/hyposensitivity and delayed information processing.

Various autism management strategies have emerged. These interventions are mainly educational, psychological, behavioural or developmental. Independent of the type of interventions, there are specific factors that increase functional skills that are commonly reported in the literature: the precocity of interventions, their individualized and structured nature, the intensity of treatment, and the construction of hierarchical and specific objectives [2]. Actions executed in different living environments allow the generalization of acquisitions. A partnership with families seems essential to promote their active participation in the education of their child.

Several interventions using play therapy and/or requiring parents’ involvement have been established to increase developmental abilities. The early start Denver model (ESDM) is a behavioural and developmental intervention based on play therapy for young children between 12 and 60 months. A recent meta-analysis based on 12 studies and 640 children receiving ESDM concluded an improvement in cognition and language [3]. However, compared to other approaches, this intervention did not show increased benefits on the symptomatology of autism, adaptive behaviour, social communication or restrictive/repetitive behaviours [3]. Joint attention, symbolic play engagement and regulation (JASPER) is an approach based on a combination of developmental and behavioural principles that targets the foundation of social communication for ASD children aged 12 months to 8 years [4]. A particularity of such a model is that is to be implemented by parents as well as clinicians or teachers. The results established on longitudinal follow-up study showed improvements in joint engagement, social communication, and emotion regulation [4]. Parent-mediated social communication therapy for young children with autism (PACT) is an approach aiming to optimize parental interactive behaviour to improve parent-child interaction and increase child communication through filmed parent-child play sessions [5]. This therapy assists parents in recognizing and responding to the child’s contextual, nonverbal and verbal signals and in interpreting the child’s intention, leading to an increase in child initiations and an enhancement of parent-child reciprocity and positive repertoires of dyadic interaction. RCTs showed that PACT intervention reduced autism symptoms in young children more than treatment as usual as far as 5.75 years after the last PACT intervention [5, 6]. These results suggest that developmental play therapy approaches involving parents have a beneficial effect on autism symptoms and the communication of ASD children.

Other forms of play therapy programs have been supported by preliminary studies. The Son-Rise program was developed to improve communication skills and learning abilities using play between family members and children with ASD. A study with 49 children (mean age 5 years) showed more (parent-reported) progresses, 4 to 6 months after a five-day parent-training course in Son-Rise Program intervention, with greater gains associated with greater hours of treatment per week [7]. A controlled trial found that 6 children (aged 4 to 6 years) who received 40 hours of the Son-Rise program for a week had a significant increase in the rate of eye contact and social contact compared to a control group who received no intervention [8]. However, the limited number of children and absence of randomization does not allow us to conclude on the efficacy of the Son-Rise program. Floortime is an evidence-based approach promoting human development that is used with children, young adults, and even adults, particularly with ASD [9]. This developmental play therapy favoured interaction between children and parents. A RCT with 32 children 2 to 6 years old, showed that adding parental intervention at an average of 15.2 hours/week for 3 months resulted in significant gains in functional emotional development and reduced severity symptoms of preschool ASD children [10]. “Exchange and Development Therapy” (EDT), which was developed in the 1990s by Lelord in France, favours one-to-one interaction between the child and a practitioner using play therapy [11]. A study with 35 children aged 2,5 to 7 years, suggested an improvement of the quality of exchange, reciprocity, communication and adaptation, 9 months after implementing this intervention [11].

The 3i intervention is mainly a developmental approach. It takes its name from three specific characteristics of the method: intensity, individuality, and interaction. It was inspired by the Son Rise Program® and by nature close to exchange and development therapy [12]. The 3i method has a similar framework as Son Rise play therapy: a quiet playroom, a one-to-one setting, and joining the child to induce interactive exchange. The 3i intervention has been enriched by a more developmental, sensory and less empirical guiding view. In general, the method relies on some currently accepted scientific bases, like the need for person with autism, to reduce the sensorial complexity, or the benefits of child imitation to favour contact through play [13–15]. These steps favour integration into school and social life. The 3i has been implemented in France and in Poland, showing that the intervention can be set up independently of the developer of the method [16, 17]. To help this implementation, the description of the 3i method has already been published, and a handbook containing all available information is currently under preparation [18].

A prospective exploratory study was carried out to assess benefits for patients [19]. Twenty “3i” patients were followed-up with for 2 years, and their developmental skills progression towards communication, socialization and imitation was assessed using Vineland Adaptative Behavior Scale (VABS) [20], Nadel’s imitation scale, and the PsychoEducational Profile Revised (PEP-R); ASD severity was assessed by the Childhood Autism Rating Scale (CARS) and the Autism Diagnostic Interview-Revised (ADI-R). The data showed a significant improvement in socialization and communication (according to VABS with parents) and imitation (Nadel’s imitation scale, assessed by a psychologist), and a significant increase in perceptual and cognitive skills according to PEP-R, as well as a decrease in autism severity based on CARS and ADI-R. These results suggest that the 3i intervention allowed improvement of relational skills that were initially very impaired in this population (mean developmental age according VABS socialization and communication scores was 12,8 months and 15,8 respectively, for a mean chronological age around 5 years). Another retrospective study was conducted on another sample of 120 patients and showed significant improvement of communication and imitation skills after 2 years of the 3i intervention based on psychologist observations through a qualitative approach [21]. Moreover, a national French survey conducted on parents of children managed by the 3i intervention reported improvement in terms of communication skills and interaction within the family [22]. Altogether these results, through either psychological assessments or parent structured reports, suggest that the disabilities associated with autism spectrum disorders might decrease during the 3i intervention, although the lack of blind assessment and control group are biases that must be emphasized.

To better assess the progress of children during the 3i intervention on a larger sample, it was decided that “3i” psychologists would systematically perform CARS and ADI-R at the beginning of the intervention and 2 years later. The aim of the present study was to assess the evolution of a large sample of children with ASD using these scales.

## Methods

### Study design

This retrospective study evaluated changes in CARS and ADI-R scores in a cohort of children with autism spectrum disorder (ASD) receiving the 3i intervention between 2013 and 2018. These evaluations were systematically performed for each patient at the implementation of the intervention (T0) and then after 2 years (T1). These ASD scales are recognized by the international scientific community and frequently used in the management of autism [23]. Although the ADI-R is usually used to diagnose ASD, here we used the *current* version, that can be used to assess the evolution of the severity of ASD [24].

### Selection of the sample

All legal guardians of children managed with the 3i method were notified that CARS and ADI-R scores would be used in a study. Their non-opposition for the use of these data was listed in June 2020 according to articles 13 and 14 of the general data protection regulations and to the reference methodology MR-004 (retrospective research on patients’ files).

**Fig. 1:**
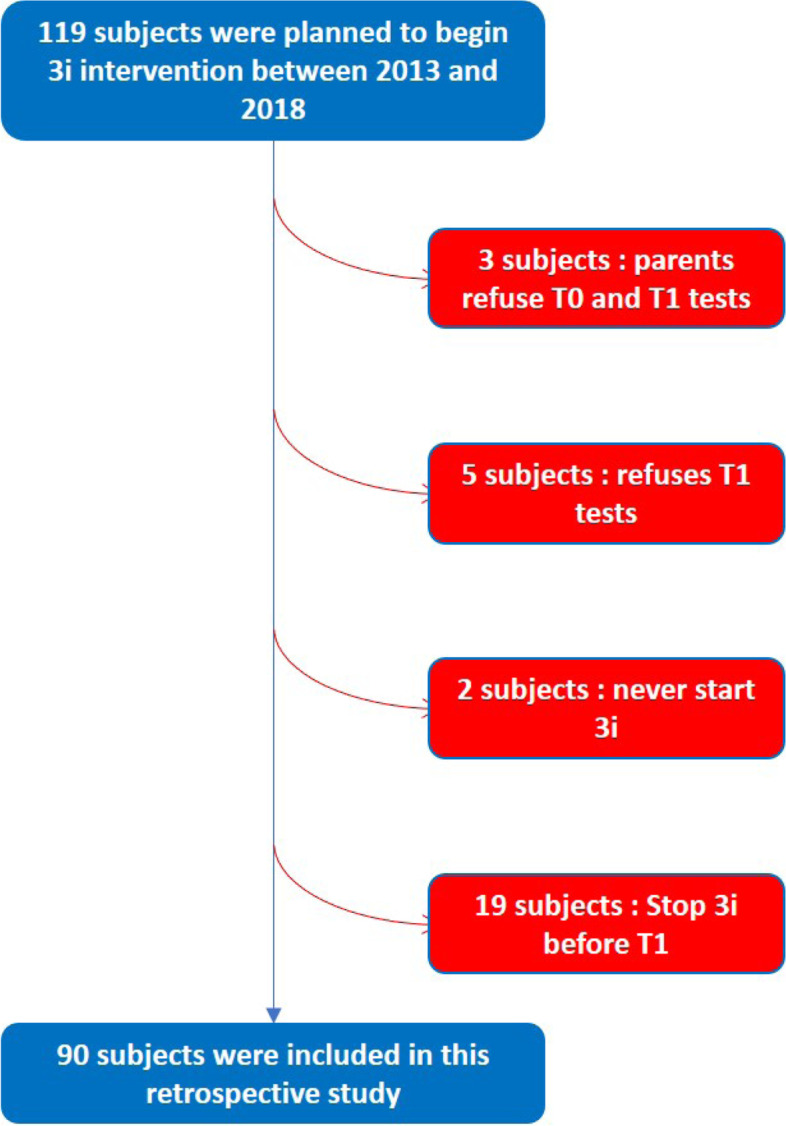
Flow chart of the study

The files of all subjects (*N* = 119) who began the method between January 1st, 2013, and January 1st^,^ 2018, were collected. Most of the participants received the intervention at home, wherever in France, whereas three of them followed the method in the Lud’eveil centre in Courbevoie (Courbevoie municipality had created a centre providing play-rooms for those families who can’t find a place to set one in their own home). At this stage, there is no evidence suggesting a difference in the effectiveness of the method between participants in home care or in educational centres [19]. Three participants who followed 3i refused evaluation. Five families refused reevaluation at FU, and two never started the 3i intervention. Finally, 19 patients stopped 3i therapy before the end of the two -year period. Thus, a total of 90 participants were included in this study (see flow chart in Fig. 1). The 19 oldest files were those of children previously included in the prospective study conducted on patients who began the intervention between January 2013 and December 2013. In this published study, patients were assessed with CARS and ADI-R but also with PEP-R and VABS at the beginning and after 2 years of 3i intervention [19].

### 3i method

The 3i method was implemented as previously described [19]. 3i is an interactive, intensive and individual intervention for autistic children. This holistic approach is built on a developmental approach using play therapy and the child-adult relationship (interactivity). The 3i method is delivered during the week (usually between 20 and 30 hours per week) by parents and external volunteers, previously selected and trained by the Autisme Espoir Vers l’Ecole (AEVE), the association in charge of the development and the establishment of the 3i. Each 3i session (with one adult for one child: individuality) lasts 90 minutes and is recorded. The recommended total time is 30 hours per week (intensity). The parents could be involved in some cases in one or two weekly play sessions and in any case in the monthly supervision sessions conducted by a 3i supervisor psychologist. During these meetings, the psychologist manages the parents’ and volunteers’ team, giving advice according to participants’ questions, ensuring parents’ and volunteers’ action consistency and compliance with the 3i method. This psychologist also analyses the child’s progress and leads therapy evolution according to child development.

The 3i playroom is usually set up in the child’s house and adapted to the sensory specificities of children with ASD. The recommended size of the playroom is approximately 10 m2, giving the child a space with visible borders. The lighting must be attenuated, and the sounds are muffled through suitable flooring. The room’s equipment includes shelves, out of reach of the child, where objects visible to the child will be stored, such as a mirror, a swing and other objects used to acquire a physical perception of oneself.

### ASD initial assessment

Seventy-one (78.9%) participants had previously received a clinical diagnosis of ASD or pervasive developmental disorder (PDD) by a psychiatrist before the 3i intervention. ADI-R assessment was completed for all selected participants and confirmed the previous independent ASD/PDD diagnosis for these 71 participants and the clinical suspicion of ASD diagnosis for the 19 other participants who were not previously diagnosed. From the participants’ chart, we also extracted age, sex and the existence of a comorbidity (e.g., seizure, genetic syndrome or ADHD) at baseline.

### Outcome measures

In our study, ADI-R scores and diagnostic categories were also used as outcome measures. ADI-R is a diagnostic tool that has been internationally approved. All ADI-R ratings were carried out by a fully trained psychologist. Since the study was a retrospective one, the psychologist was not blinded for the time of assessment. The ADI-R is a semistructured standardized interview administered by qualified clinicians to the parents or caregivers of persons referred for suspected ASD [25]. The ADI-R comprises 93 items in three areas: communication and language, quality of reciprocal social interactions, and so-called restricted, repetitive and stereotypical behaviours. Forty-two ADI-R items are systematically combined according to an algorithm to produce an autism diagnostic based on ICD-10 and DSM-IV criteria. A score is calculated for each of the “interaction”, “communication” and “repetitive” domains. The domain is considered typical if the score is strictly less than a specified threshold. The threshold of the score is 9 for the “interaction” domain, 8 for the “communication” domain if the subject is nonverbal (or 7 if the subject is verbal), and 3 for the “repetitive” domain. If all areas were typical, the patient was classified “without pervasive developmental disorder”. If one or two domains are typical, the subject is classified as “atypical autism.” Finally, if no domain is rated typical, the subject is classified in the “typical autism” category.

CARS is the standardized instrument most widely used in the autism severity assessment process [26]. It is a semistructured interview which deals with the 15 following areas: social relations, imitation, emotional responses, use of the body, use of objects, adaptation to change, visual responses, auditory responses, taste-smell-touch (responses and modes of exploration), fear and anxiety, verbal communication, nonverbal communication, level of activity, intellectual level and homogeneity of intellectual functioning and general impression. Subjects with scores between 30 and 36.5 are classified as having “mild autism”, and subjects with a score of 37 or higher are classified as having “severe autism”. Patients with a score strictly less than 30 are classified in the “non-autistic” zone. CARS scores were rated here by trained psychologists on the basis of 3 to 4 play sessions and a meeting with the parents.

The results of each assessment were recorded in the patient’s personal file. Each file received a unique code that allows anonymization of the data. The quantitative and qualitative data of these evaluations were compiled with patient-specific data (specific code, age at the time of management, sex, comorbidities associated with ASD, ASD diagnosis, starting date of the intervention, etc.).

### Statistical analysis

Patients’ CARS and ADI-R scores at the beginning (T0) were compared with the respective scores at the two-year FU (T1) mark using the paired student’s t-test. The effect-sizes were calculated according to Cohen [27], 0.2 being considered a ‘small’ effect size, 0.5 a ‘medium’ effect and 0.8 a ‘large’ effect size. Additionally, the distribution of patients within the severity groups related to CARS or ADI-R scores was compared between T0 and T1 using Fisher’s exact test (Freeman-Halton expansion) [28].

To assess the weight of baseline variables in the longitudinal evolution of the ADI-R and CARS scores, a multiple linear regression model was used. The predictive variables were participants’ age, sex, presence of a known comorbidity, CARS score at T0, intervention intensity (more or less than 30 hours of 3i therapy per week) and verbal or non-verbal communication at T0 (based on the ADI-R communication initial assessment). The models were calculated as follows:

$$\mathrm{F}\left(\mathrm{ratioT}1/\mathrm{T}0\right)=\mathrm{age}+\mathrm{sex}+\mathrm{comorbidity}+\mathrm{test}\ \mathrm{at}\ \mathrm{T}0+\mathrm{Intensity}3\mathrm{i}+\mathrm{communication}.$$

A two-sample paired t-test was used to compare the T1/T0 ratio for two subgroups (more or less than 30 hours of 3i intervention) for the CARS score and each of the ADI-R scores.

Since this retrospective study did not include a control group, we aimed to compare our cohort evolution over 2 years with that described over 3 years in the Baghdadli et al. study for CARS [29]. Based on mean CARS, we performed a linear regression to obtain the slope of score evolution over time. Then, we compared this slope with the mean of the slopes calculated for each patient using a one sample t-test. *P* values were considered significant at *p* < 0.05. Statistical analyses were performed using R software version 3.4.3.

## Results

### 3i cohorts

Ninety subjects were included in this retrospective cohort, consisting of 17 girls and 73 boys (Table 1). Their average age at baseline was 5.6 ± 3.7 years. At T0, the youngest subject was 2 years old and the oldest 18 years old. The average length of time between the two assessments was 2.25 ± 0.38 years. Fourteen patients had comorbidities: epilepsy (*n* = 2), −fragile X (*n* = 3), Down syndrome (*n* = 1), tetrasomy 12p with mild hearing loss (*n* = 1), cerebellar syndrome (*n* = 1), heart disease (*n* = 1), West syndrome (*n* = 1) and ADHD (*n* = 4).

**Table 1:** Sample description at baseline

	N	Age	ADI-R Typical autism	ADI-R Atypical autism
Total	**90** (100%)	5.6 ± 3.7	77 (85.6%)	13 (14.4%)
Girls	**17** (19%)	5.3 ± 3.0	13 (76.5%)	4 (23.5%)
Boys	**73** (81%)	5.6 ± 3.9	64 (87.7%)	9 (12.3%)
Age ≤ 3 years	**34** (38%)	2.5 ± 0.5	31 (91.2%)	3 (8.8%)
4 ≤ Age ≤ 8 years	**42** (47%)	5.6 ± 1.4	37 (88.1%)	5 (11.9%)
Age ≥ 9 year	**14** (15%)	12.9 ± 2.3	9 (64.3%)	5 (35.7%)
Comorbidity	**14** (15%)	6.9 ± 3.0	11 (78.6%)	3 (21.4%)
Without listed comorbidity	**76** (84%)	5.3 ± 3.8	66 (86.8%)	10 (13.2%)
> 30 h/week of 3i	**48** (53%)	5.6 ± 3.5	43 (89.6%)	5 (10.4%)
< 30 h/week of 3i	**42** (47%)	5.4 ± 4.1	34 (70.8%)	7 (29.2%)
Verbal (T0)^a^	**31** (34%)	7.2 ± 4.0	23 (74.2%)	8 (25.8%)
Non-Verbal (T0)^a^	**59** (66%)	4.7 ± 3.3	54 (93.2)	5 (6.8%)

^a^Verbal or non-verbal communication status was defined through ADI-R communication initial assessment

According to the T0 ADI-R assessment, 13 subjects (14.4%) were classified as having “atypical autism” and 77 (85.6%) as having “typical autism.” Thus, most subjects (six out of seven) had typical autism, and even more (nine out of ten) young children under three. Moreover, two out of three subjects were nonverbal (according to ADI-R communication subscores) with higher rates among the younger patients (Table 1).

No serious adverse effects related to the intervention were reported during the 2-year period.

### ADI-R score evolution between T0 and T1

At the 2 year FU, the ADI-R interaction domain score fell from an average of 18.3 to 11.1. The ADI-R communication domain score felt from 11.6 to 8.3, and the repetitive domain score fell from 5.7 to 4.1. All scores’ drops were significant (Table 2). The average change in the three scores between T0 and T1 remained statistically significant when adjusted for the age and sex of the patient, presence of listed comorbidities, ADI-R score at T0, verbal or nonverbal communication status at T0 and weekly intensity of the 3i intervention (Table 3).

**Table 2:** Change in ASD severity after the two-years intervention

Tests	T0 Beginning 3i (mean ± SD)	T1 after 2 years (mean ± SD)	Evolution(Ratio T1/T0 in % ± SD)	Effect size (d)	*p* Value
CARS	40.3 ± 7.1	32.3 ± 7.2	−20.0 ± 14.9%	1.036	1.16E-18
ADI-R Reciprocal Social Interaction	18.3 ± 6.0	11.1 ± 5.7	−41.0 ± 25.2%	1.567	7.45E-26
ADI-R Language/Communication	11.6 ± 3.3	8.3 ± 3.8	−27.5 ± 32.9%	0.884	6.82E-13
ADI-R Repetitive Behaviors/Interests	5.7 ± 2.0	4.1 ± 1.8	−25.0 ± 31.6%	0.824	1.02E-11

**Table 3:** Weight of variables in ADI-R score change based on linear regression models

	Variable	Coefficient	SD	t-value	***P*** value
**ADI-R Reciprocal Social Interaction**	**> 30 hours per week or less**	−12.58	5.04	−2.496	0.0146^*^
	**Age**	−0.29	0.72	− 0.398	0.6914
	**Sex male or not**	0.11	6.47	0.016	0.987
	**ADI-R score at T0**	−0.09	0.51	− 0.182	0.8563
	**Verbal communication or not at T0**^a^	−14.82	6.40	−2.316	0.0231^*^
	**Listed comorbidity or not**	−1.42	7.23	−0.197	0.8445
**ADI-R Language/Communication**	**> 30 hours per week or less**	−9.88	6.77	−1.458	0.14859
	**Age**	−0.25	0.97	−0.263	0.79309
	**Sex male or not**	5.62	8.86	0.634	0.52788
	**ADI-R score at T0**	−1.32	1.09	−1.21	0.22971
	**Verbal communication or not at T0**^a^	−20.36	7.66	−2.659	0.00944^**^
	**Listed comorbidity or not**	−4.52	9.67	−0.47	0.64
**ADI-R Repetitive Behaviors/Interests**	**> 30 hours per week or less**	0.55	6.95	0.08	0.94
	**Age**	−2.32	0.99	−2.34	0.022^*^
	**Sex male or not**	2.43	8.58	0.28	0.78
	**ADI-R score at T0**	−0.67	0.56	−1.20	0.24
	**Verbal communication or not at T0**^a^	−4.69	8.79	−0.53	0.60
	**Listed comorbidity or not**	−14.75	9.77	−1.51	0.14

^a^Verbal or non-verbal communication status was defined through ADI-R communication initial assessment; **p <0.01; *p <0.05

**Fig. 2:**
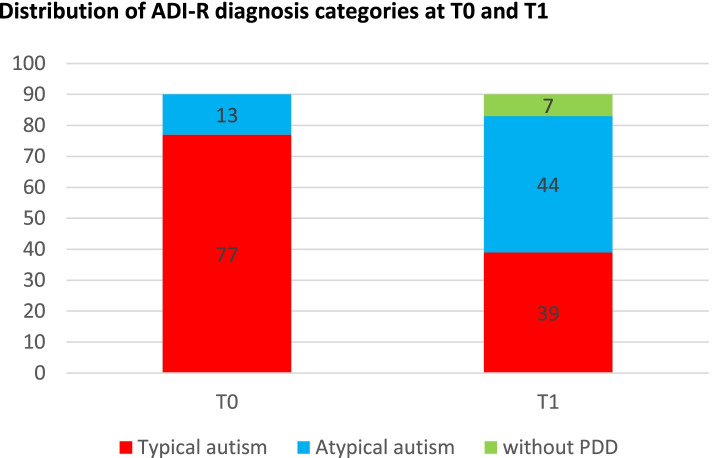
Distribution of ADI-R diagnosis categories at T0 and T1

At T1, only 39 participants were categorized as having “typical autism” compared to 77 at T0, 44 were categorized as displaying “atypical autism” at T1 versus 13 at T0, and seven participants were categorized as without PDD at T1 versus none at T0 (Fig. 2). Out of 77 participants categorized as having “typical autism” at T0, 39 stayed in this category at T1, 35 changed to the “atypical autism” category and 3 to “without PDD”. Out of 13 participants, nine stayed in the “atypical autism” category 2 years later, and four changed to “without PDD”. Thus, 42 participants out of 90 (46.7%) changed their autism severity category based on the ADI-R interview. The distribution of category change between T0 and T1 was significant based on Fisher’s exact test (*p* value = 6.77. 10^− 5^). Overall, the 3 ADI-R subscores significantly reduced and the ADI-R score category significantly changed for participants receiving 3i intervention over 2 years.

#### CARS evolution between T0 and T1

Only 80 participants had a CARS scale at T0 and T1 (8 CARS are missing at T0 and 5 CARS at T1). The mean CARS score dropped from 40.3 to 32.3, with a mean significant decrease of approximately 20% (*p* value = 1.16.10^− 18^, Table 2). The average decrease between T0 and T1 remained statistically significant when adjusted for age, sex, presence of listed comorbidities, CARS score at T0, verbal or nonverbal communication status at T0 and intensity of the 3i intervention per week (Table 4).

**Table 4:** Weight of variables (coefficient and *p* value) in the CARS score change based on linear regression models

Variable	Coefficient	SD	t-value	***P*** value
**Intensity > 30 hours per week or not**	−3.1551	3.3548	−0.94	0.3501
**Age**	0.5723	0.4688	1.221	0.2261
**Sex male or not**	4.7679	4.0823	1.168	0.2467
**CARS score at T0**	−0.6568	0.2699	−2.433	0.0175^a^
**Verbal communication or not at T0**^a^	−8.9058	4.2459	−2.098	0.0395^a^
**Listed comorbidity or not**	5.7237	4.6317	1.236	0.2206

^a^Verbal or non-verbal communication status was defined through ADI-R communication initial assessment

**Fig. 3:**
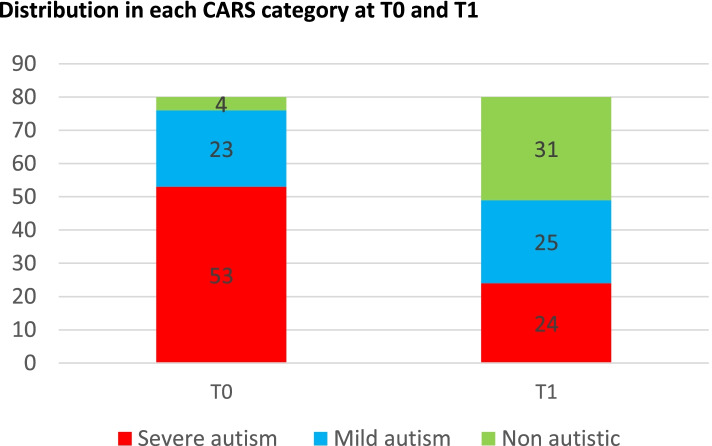
Distribution in each CARS category at T0 and T1

Two years after 3i implementation, the number of participants in the “severe autism” category dropped from 53 to 24, increased from 23 to 25 in the “mild autism” category and increased from 4 to 31 in the “non-autistic” category (Fig. 3). Thus, 44 out of the 80 patients changed their CARS category after 2 years of 3i intervention (*p* value = 1.43. 10^− 4^, Fisher exact test). Twenty-three of 53 of the “severe autism” participants at T0 stayed in this category, and the others changed to “mild autism” (17/53) or non-autistic (13/56). Of the 23 patients categorized with “mild autism” at T0, 14 changed to the non-autistic category, 8 remained in the “mild autism” category and 1 felt to the severe autism category. The four participants categorized as non-autistic at T0 remained in this category at T1. In conclusion, there was a significant decrease in the mean CARS score between T0 and T1, and over half of the participants changed to a lower category of ASD severity.

#### Linear multiple model

To better understand whether some patient’s or environmental traits could affect the evolution of ADI-R and CARS scores, multiple linear models were created for ADI-R and CARS changes to assess the weight and significance of variables as described in the Materials and Methods section. Sex and comorbidities had no significant impact on the decrease observed for the 3 ADI-R subscores and CARS score.

However, the ADI-R interaction score change was partially dependent on the method intensity (Table 3). Participants with more than 30 hours of 3i intervention per week showed a significantly better progression of their ADI-R interaction score. Additionally, participants with a verbal communication ability at T0 had a significantly better progression of the ADI-R interaction and ADI-R communication subscores compared to nonverbal participants (Table 3). Finally, older participants displayed a significantly larger decrease in the ADI-R stereotypies subscore (Table 3).

The CARS score evolution was associated with the CARS score at T0 and verbal communication status at T0. Participants with higher CARS scores (higher autism level) at T0 had a significantly better decrease in their CARS score at T1. In addition, participants with verbal communication abilities at T0 showed a significantly better decrease in their CARS score than nonverbal participants (Table 4).

## Discussion

### Effects of 3i method on autism severity

In the current study, we followed 90 children with ASD during 2 years of intervention with the developmental 3i method. Mean CARS scores and ADI-R subscores (interaction, communication, repetitive behaviour) decreased significantly. In addition, half of the participants switched to a lower category of ASD severity based on both the CARS and ADI-R.

Due to the absence of a control group (see Limits of the study), it is important to know whether the change to a lower ASD diagnostic category severity according to ADI-R observed in 42 out of 90 participants (46%) was linked to 3i intervention. The ADI-R has been used in several studies to assess the evolution of patients [24, 30–34]. Lord and colleagues, for example, measured the ADI-R score evolution in 172 children followed-up with usual treatment between two assessments realized at two and 9 years of age [35]. They showed that the three mean ADI-R subscores remained stable, with only a few patients who were classified as having pervasive developmental disorder not otherwise specified (PDD-NOS) at 2 years old and became classified as autistic at the age of nine. Rondeau and colleagues performed a meta-analysis to assess the stability of the ASD diagnosis related to ADI-R and ADOS [36]. In this meta-analysis, based on eight studies and 444 participants, patients underwent various “as usual” interventions, including speech therapy or educational therapy, with heterogeneous intensity levels. Evaluations repeated between 1 and 7 years after the first assessment showed that autism diagnostic (AD) was more stable than PDD-NOS diagnostic (Additional data, Table 1). Indeed, in Rondeau and colleagues, out of 444 participants, 65% remained categorized with their previous ASD diagnostic category (additional data, Table 2); 24% changed to a lower diagnostic severity (AD to PDD-NOS or non-ASD) and 11% to a more severe severity (PDD-NOS to AD). In this Rondeau’s aggregated cohort, mean age at the beginning of the intervention was 28,6 months, and 72,5% had typical autism whereas mean age of our cohort was 66 months and 77% had typical autism. Children of Rondeau’s cohort were younger so that they should have made more progresses and should have more changed of category than those of our cohort. However, in the 3i cohort, 53% of participants remained in their previous diagnostic category, 47% changed to a lower diagnostic category severity, and none changed to a more severe one (additional data, Table 2). Although it is difficult to draw conclusions, these data may suggest a better outcome regarding ASD diagnosis in the 3i cohort than in Rondeau’s aggregated cohort.

Regarding CARS, we compared the current results with those of the Baghdadli et al. cohort, the largest French longitudinal cohort on autism [29]. In the latter, CARS was used to assess the evolution of 152 children with ASD, mainly followed-up in child and adolescent psychiatry departments and autism evaluation clinics located in public hospitals. The follow-up lasted for 10 years [29]. We compared the CARS evolution of Baghdadli’s cohort with that of our 3i cohort. Children in Baghdadli’ cohort were French with a mean age of 57 months at the first evaluation, with 85% of typical autism, so that they were not so far from those of our sample. Around 9% had comorbidity versus 15% in our cohort. The slope of CARS decreased 4.28-fold more in 3i children than in Baghdadli’s cohort (*p* value < 0.0001, additional data).

Since these studies were conducted on different samples and the data were obtained from different studies with their own design, we must be very careful in comparing these data. However, this may suggest that autism severity could decrease more in the 3i cohort compared to that reported in previously published studies with as usual treatment. Thus, we show these data in additional tables and figures for illustrative purposes only.

### Intensity of the 3i method may affect outcomes

Intensity is known to be associated with better outcomes [37]. This was the case in the current study: a greater intensity of intervention was significantly associated with a greater decrease in the ADI-R interaction score. This association is important because it suggests that the observed improvement is, at least for some components, due to the intervention rather than to a spontaneous improvement over time. A dose/response effect has already been described in a smaller previous study on 19 ASD participants with 3i intervention [19]. In this latter prospective study, all outcomes based on PEP-R, VABS, CARS, ADI-R, and an imitation scale showed a significant improvement after 2 years of intervention with the 3i method. In addition, the increase in VABS socialization score was positively correlated with the total number of hours of 3i intervention, suggesting that socialization ability improved because of the multiple and intensive interactions provided through the 3i method. This dose/response effect seems to be reproduced in the current study concerning the interaction subscore of the ADI-R. Improvement in developmental skills was also observed in a cohort of 49 ASD children treated with the Son-Rise program (SRP) more than 22 hours per week, less than 20 hours per week, or no SRP [7]: parents who administered Son-Rise Program intervention reported improvements in communication, sociability, and sensory and cognitive awareness in their children, with greater gains associated with greater hours of treatment per week. However, heterogeneity of developmental skills between the two SRP groups did not allow us to conclude a robust intensity effect of SRP.

### Effects of 3i intervention on autism symptoms

Improvement in communication and socialization scores in this study appears to also be consistent with another retrospective study on 120 children who received 2 years of 3i intervention [21]. This previous study used a home-made scale scoring some key-behaviors in 6 domains through written reports from supervision sessions; it showed a significant improvement in all the six explored domains (imitation, socioemotional regulation, gaze quality, nonverbal expression, verbal expression, and verbal comprehension) with a greater improvement in imitation and nonverbal communication.

Together, these three studies suggest that 3i intervention may help children gain developmental abilities in the social interaction and communication domains that are the core of autistic symptoms. It may make sense that the 3i intervention could preferentially reduce handicaps related to interaction as measured by ADI-R interaction or VABS socialization. Indeed, the 3i method proposes intensive play therapy with three to four different adults per day, following child initiations. Given this child-centred interactive focus, we can speculate that the first improvement is linked to the motivation and ability to meet other persons and interact through nonverbal cues, whereas progress in language may be significant to a lesser extent because they will emerge subsequently as the child becomes more willing to interact, communicate, make eye contact and share attention with others, which are prerequisites to develop verbal communication. Longer follow-up studies could contribute to exploring this point. Estes and colleagues (2015) demonstrated continued positive impacts on development 2 years after their individual interactive intervention (ESDM) ended [38].

### Other predictive factors mediating reduction of ASD severity

In addition to method intensity, we examined whether other factors could mediate the observed decrease in scores. Intellectual quotient and verbal IQ (VIQ) language development skills, nonverbal communication skills and motor skills have already been associated with optimal ASD outcomes [39–43]. We wondered if the age of the participants, sex, severity of ASD, or comorbidity naturally could have an impact on their behavioural and developmental skills. Despite using multiple linear models, we were unable to demonstrate an impact of age, sex or comorbidity on the observed decrease in CARS and ADI-R scores. In contrast, being categorized as a verbal subject by the ADI-R assessment at the beginning of the 3i intervention was associated with better outcomes concerning CARS and ADI-R interaction and communication subscores (Tables 3 and 4). As it is known that a better intellectual level, or developmental quotient, is a factor of better prognosis [29, 44], it is tempting to propose that patients with higher verbal skills should perform better with 3i than those with lower verbal skills. Higher communication skills at the beginning of the 3i intervention may facilitate interaction with adults in the playroom and accelerate the decrease in ASD severity reflected by the CARS score and ADI-R’s interaction and communication subscores.

Indeed, several studies have shown that interventions involving parents have the potential to increase benefits by creating consistent opportunities for children to practice skills in diverse contexts [45]. Other approaches based on individual interactive play, particularly with parental involvement, have been shown to favour patient outcomes, such as DIR floortime [10], Son-Rise [8], PACT [5] or ESDM [46]. These results suggest that a one-to-one, interaction-centred intervention with parents or caregivers using play therapy may enhance interaction, favour language skills of ASD children and decrease symptom severity. The 3i method involved volunteers (three to four per day) to perform intensive play therapy to children with ASD. Further measurement of the patient/adult interaction level during the 3i play session could help explore this issue.

This intensive one-to-one interaction with adults during play also aims to favor imitation. Imitation is known to allow the development of socio-communicative skills such as joint attention, intention of understanding and social reciprocities [47, 48]. In our previous prospective study with 19 ASD children, the Nadel imitation score increased significantly by 49% after 2 years of 3i methods [19]. We hypothesize that in this study, the 3i method improved imitation and favoured an increase in communication and social interaction skills with a reduction in ADI-R-related subscores.

Altogether, the intensive one-to-one interaction with adults during play as organized in the 3i method could trigger the development of communication skills in children to favour a decrease in ASD severity.

### Limits of the study

The current study suffers from many limitations so that this study provides preliminary evidence, that must be supported through further studies. First, the study lacks a control group, and assessments were not blind to the received method, as all patients in this cohort followed the 3i method. However, the positive association between the decrease in ADI-R current score and intensity suggests an intrinsic effect of the 3i intervention, although there also could be a bias in parent reporting. Also, real efforts have been made to compare our results with those of Baghdadli’s cohort and of aggregated cohort of Rondeau’s meta-analysis.

Second, off the initial 119 subjects sample, 19 dropped out the intervention before 2 years and were not included. We can’t exclude the hypothesis that some of them had a lower response to the intervention.

Third, time of CARS assessments was non-blinded, and ADI-R are based on parental report which may be biased.

Fourth, there was no baseline or outcome IQ or developmental assessments, such as PEP-R or Vineland, which is frequently used in intervention studies [19, 32, 38, 46, 49] since it is the best instrument to assess changes in autism [50] and allows assessment of the developmental quotient. However, this study is a retrospective one and at the time of this study, VABS was generally not used to assess children at the beginning of intervention and 2 years after. Today, this assessment have been included in the routine evaluations of all children receiving this intervention, so that further studies will include VABS assessment.

Also, in this method, a high intensity of intervention is recommended, so that the patients who received the most hours during the 2 years were those who better applied the treatment recommendations. It could be a bias to the interpretation that the intensity of the intervention favors a better outcome. However, it is not a bias to conclude that the method was responsible for the observed progresses.

Sixth, it could be of interest to use a parent-child interaction scale, as in Pickles et al.’s study [5]. In addition, as the 3i method tends to reduce ASD severity and favour better interactions but also imposes parental time and involvement, it should be interesting to assess the parents’ quality of life and parental stress to better understand benefits for familial daily life. Finally, the impact of the 3i method on children’s lives, particularly their ability to attend school, should be included in future work.

## Conclusion

This study analysing 90 children suggests that intensive individual interactive (3i) play therapy with parents and volunteers for 2 years may allow a significant reduction in ASD severity with improvements in interaction, communication, and repetitive behaviours. These results are in line with those obtained in previous studies [19, 21] and should be confirmed in further studies. Assessment of parental and patient quality of life should be performed in future studies that should include a control group.

## Supplementary Information

**Additional file 1: Additional Table 1.** Distribution of diagnosis categories at T1 and T2 in our study compared to that of the Rondeau et al. meta-analysis. **Additional Table 2.** Ratio of change of diagnosis category between T1 and T2 in our study compared to that of Rondeau et al. meta-analysis. **Additional Figure.** CARS evolution in our study compared to that of the Baghdadli French cohort study.

## Data Availability

The datasets used and/or analysed during the current study are available from the corresponding author on reasonable request.
