# Supplementary material for: Two-year follow-up of 90 children with autism spectrum disorder receiving intensive developmental play therapy (3i method)
Source: BMC Pediatr. 2022 Jun 28;22:373. doi: 10.1186/s12887-022-03431-x (PMC9238102; doi:10.1186/s12887-022-03431-x)
Supplement: Supplementary file 1 — Additional file 1: Additional Table 1. Distribution of diagnosis categories at T1 and T2 in our study compared to that of the Rondeau et al. meta-analysis. Additional Table 2. Ratio of change of diagnosis category between T1 and T2 in our study compared to that of Rondeau et al. meta-analysis. Additional Figure. CARS evolution in our study compared to that of the Baghdadli French cohort study. [file 12887_2022_3431_MOESM1_ESM.docx]

**Additional data**

**Additional Table 1: Distribution of diagnosis categories at T1 and T2 in our study compared to that of the Rondeau et al. meta-analysis**

|  | ASD severity test at T1 | | | ASD severity test at T2 | | |
| --- | --- | --- | --- | --- | --- | --- |
|  | AD | PDD-NOS | Non ASD | AD | PDD-NOS | Non ASD |
| 3i (This study) | 77/90 (85,6%) | 13/90 (14,4%) | 0/90 (0,0%) | 39/90 (43,3%) | 44/90 (48,9%) | 7/90 (7,8%) |
| Rondeau *et al.*, 2010 | 322/443 (72,5%) | 122/443 (27,5%) | 0/443 (0,0%) | 293/443 (66,1%) | 90/443 (20,3%) | 60/443 (13,5%) |

**Additional Table 2: Ratio of change of diagnosis category between T1 and T2 in our study compared to that of Rondeau et al. meta-analysis**

|  | Change for a lower ASD severity category between T1 and T2 | Stability in ASD severity between T1 and T2 | Change for a higher ASD severity category between T1 and T2 |
| --- | --- | --- | --- |
| 3i (This study) | 42/90 (47%) | 48/90 (53%) | 0/90 (0%) |
| Rondeau *et al.*, 2010 | 107/443 (24%) | 288/443 (65%) | 48/443 (11%) |

AD: Autism disorder; PDD-NOS: Pervasive Developmental Disorder Not Otherwise, Specified; Non ASD: Non-Autist Spectrum Disorder

**Additional Figure: CARS evolution in our study compared to that of the Baghdadli French cohort study**

**
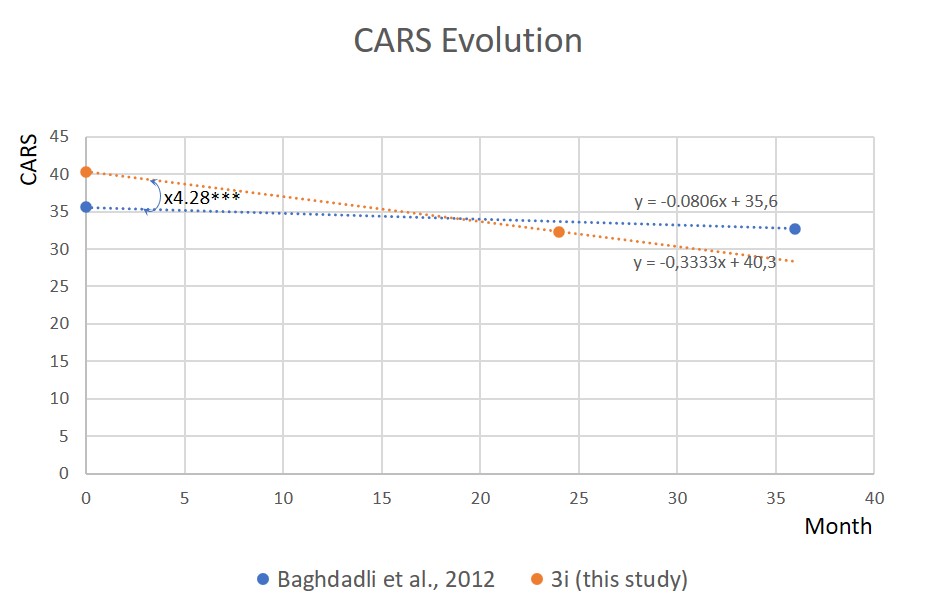
**

Evolution of the CARS scores in children following 2 years of 3i (this study, red) or assessed in the Baghdadli et al., 2012 study (blue). The mean of the related scores is represented in the diagrams. The resultant slope was calculated and is shown in the diagrams. To test significant differences between studies, the slope of each 3i child was calculated and compared to the slope of the Baghdadli et al., 2012 results using a one sample t-test. The ratio of the slope is represented in the diagram. *** p value<0,001, ** p value<0,01, * p value<0,05.
